# Supplementary material for: Age-Dependent Hippocampal Proteomics in the APP/PS1 Alzheimer Mouse Model: A Comparative Analysis with Classical SWATH/DIA and directDIA Approaches
Source: Cells. 2021 Jun 24;10(7):1588. doi: 10.3390/cells10071588 (PMC8304546; doi:10.3390/cells10071588)
Supplement: Supplementary file 1 [file cells-10-01588-s001.zip › cells-1231658-supplementary.pdf]

Supplementary Materials

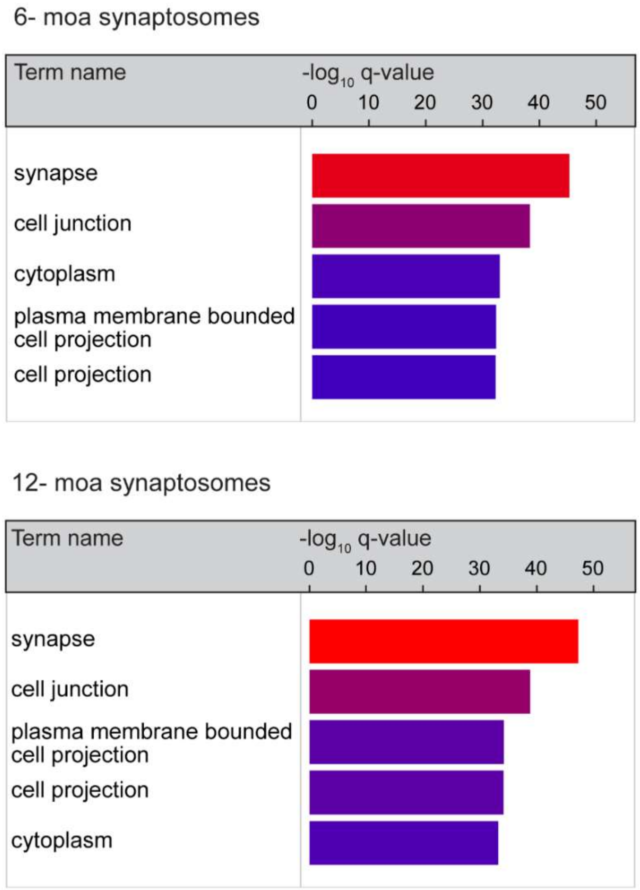

Figure S1. Enrichment of synaptic proteins in the synaptosomal sample preparation. GO-enrichment analysis of all proteins detected in the 6- and 12-moa synaptosomal datasets under investigation in the current study. Total brain expressed genes is used as background.

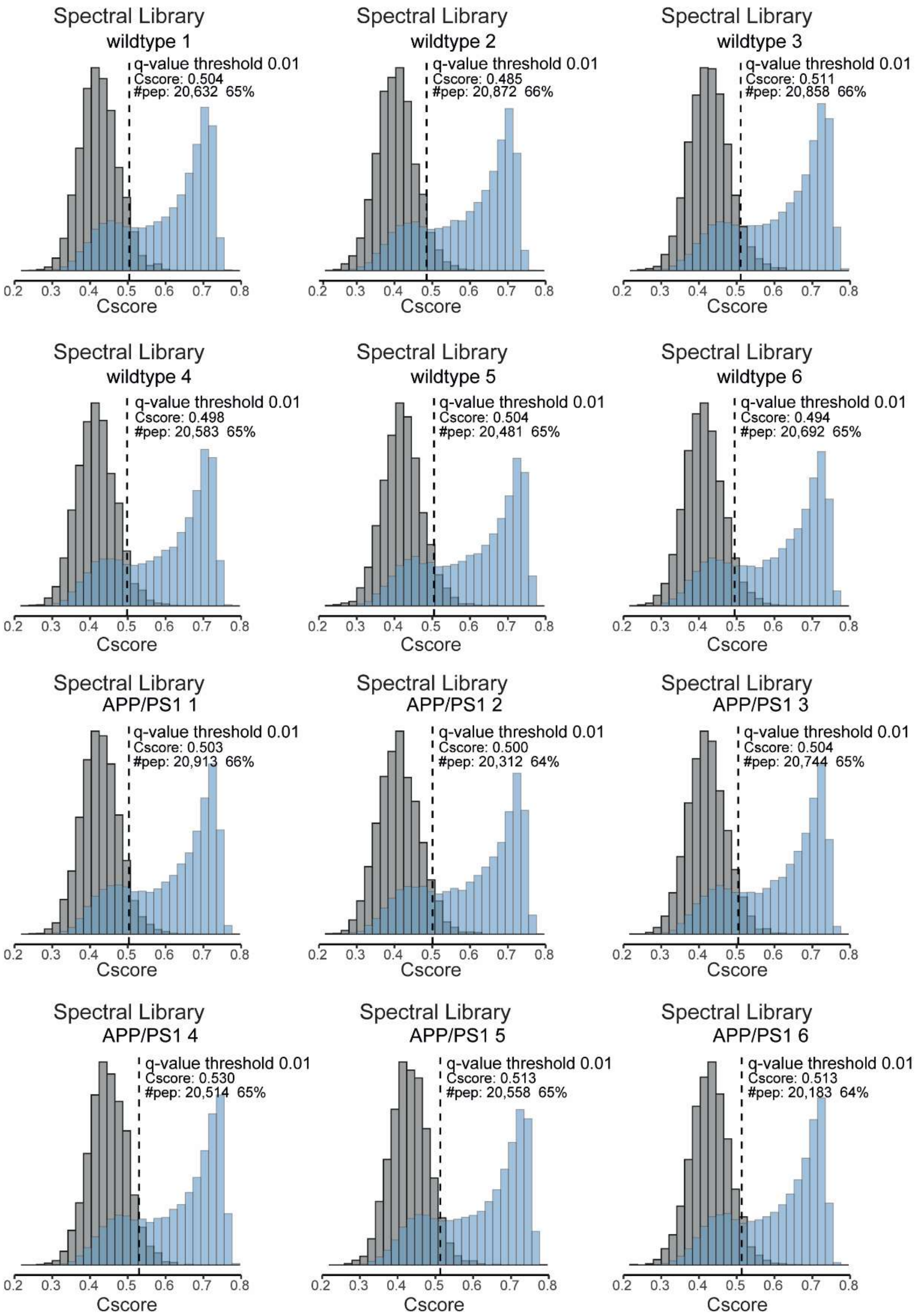

Figure S2. Target (blue) and decoy (grey) confidence score distributions of all 12-moa samples searched against the DDA-based spectral library. The confidence scores (cscores) indicate the level of confidence the software had in the identification of peptides in the raw SWATH data. The q-value confidence threshold of 0.01 is shown as a dotted line, and the associated cscore and number of peptides quantified above this threshold are reported.

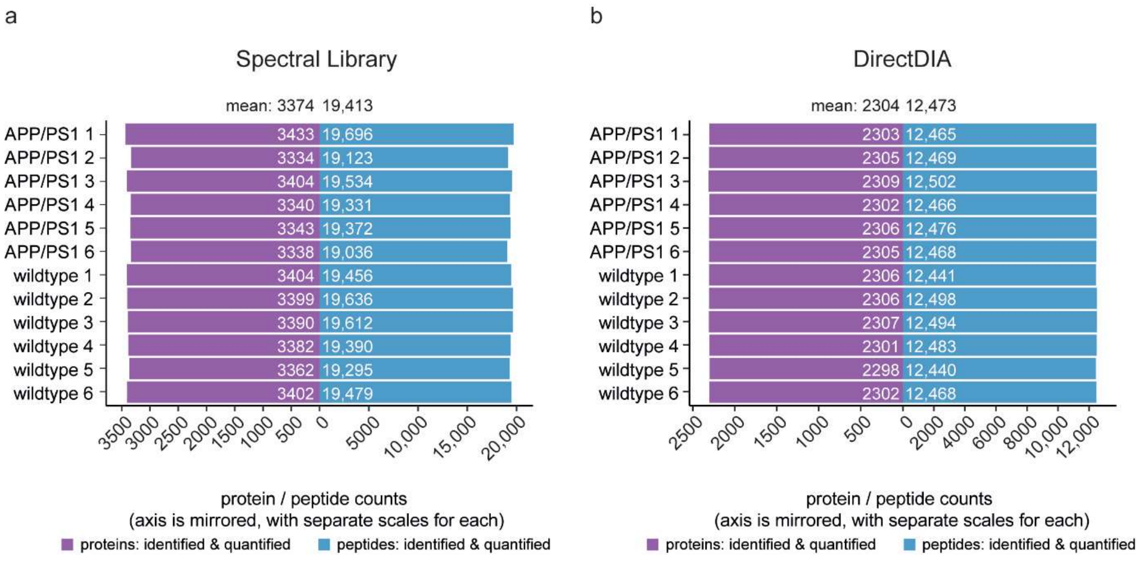

Figure S3. Number of identified and quantified proteins and peptides with a q- value ≤ 0.01 for identification. (**a)** Number of proteins and peptides identified using the DDA-based spectral library are shown on average and per individual sample; (**b**) Analogous to panel a, number of proteins and peptides are shown on average or per individual sample, identified using directDIA.

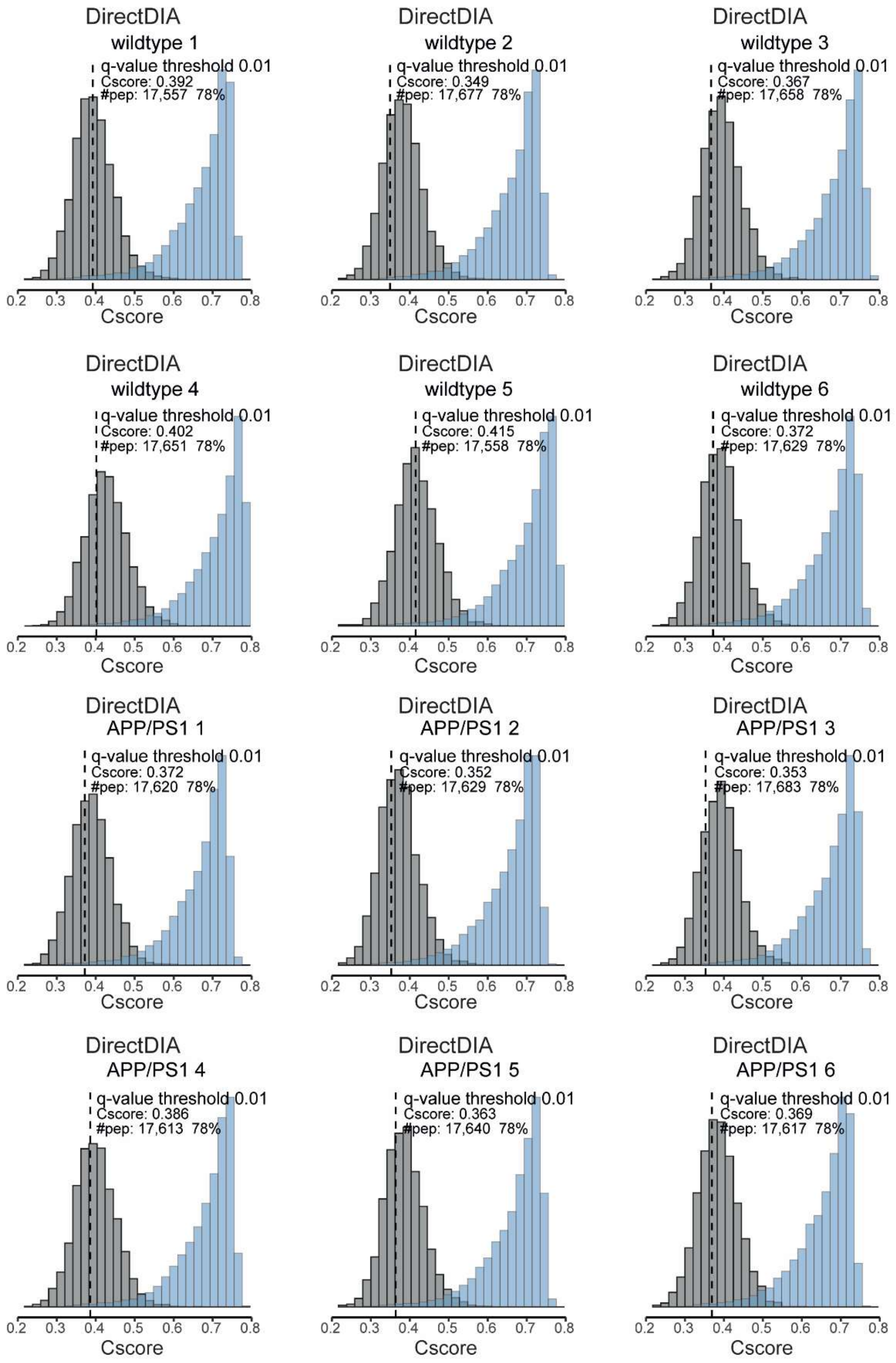

Figure S4. Target (blue) and decoy (grey) confidence score distributions of all 12-moa samples searched using directDIA. The confidence scores (cscores) indicate the level of confidence the software had in the identification of peptides in the raw SWATH data. The q-value confidence threshold of 0.01 is shown as a dotted line, and the associated cscore and number of peptides quantified above this threshold are reported.

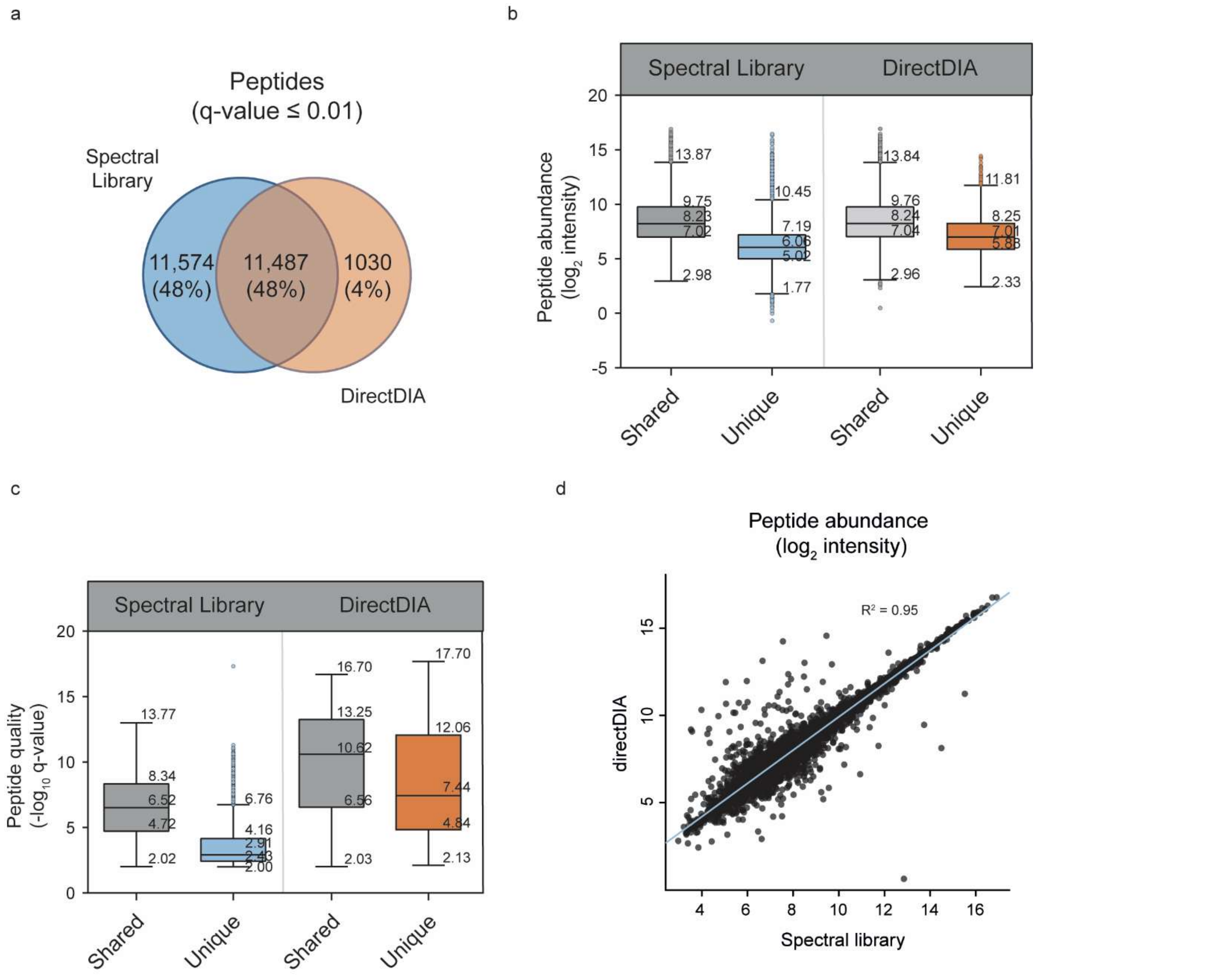

Figure S5. Characterization of unique and shared peptides identified using the spectral library or directDIA data searches. (**a**) Number of shared and unique identified peptides. (**b**) Abundance of shared and unique peptides identified using the spectral library. (**c**) Quality of shared and unique peptides identified using the spectral library. (**d**) Correlation in intensity between peptides identified using the spectral library or directDIA.

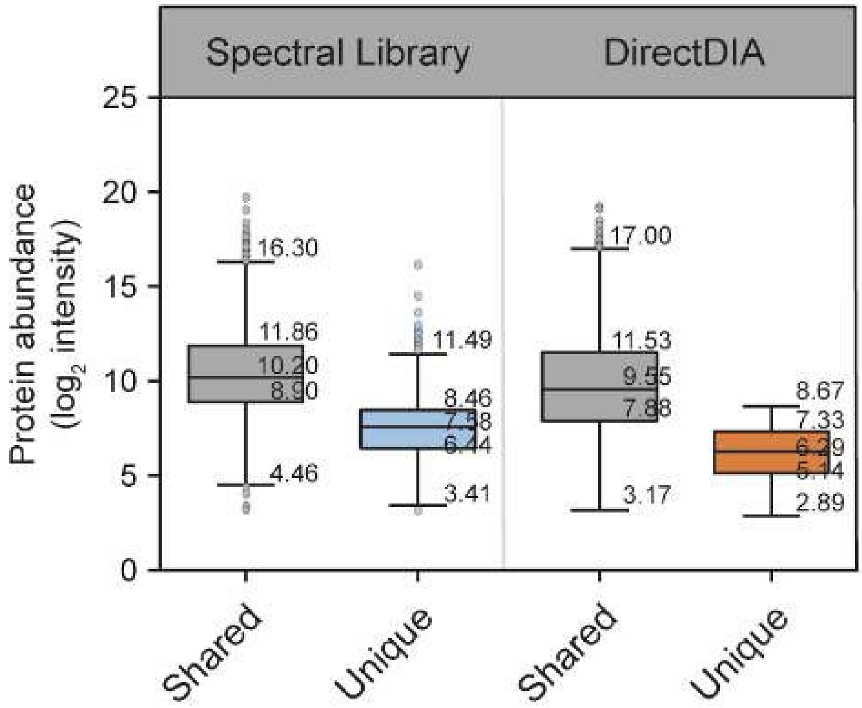

Figure S6. Abundance of shared and uniquely identified proteins using the spectral library or directDIA after filtering high quality peptides for differential expression analysis.

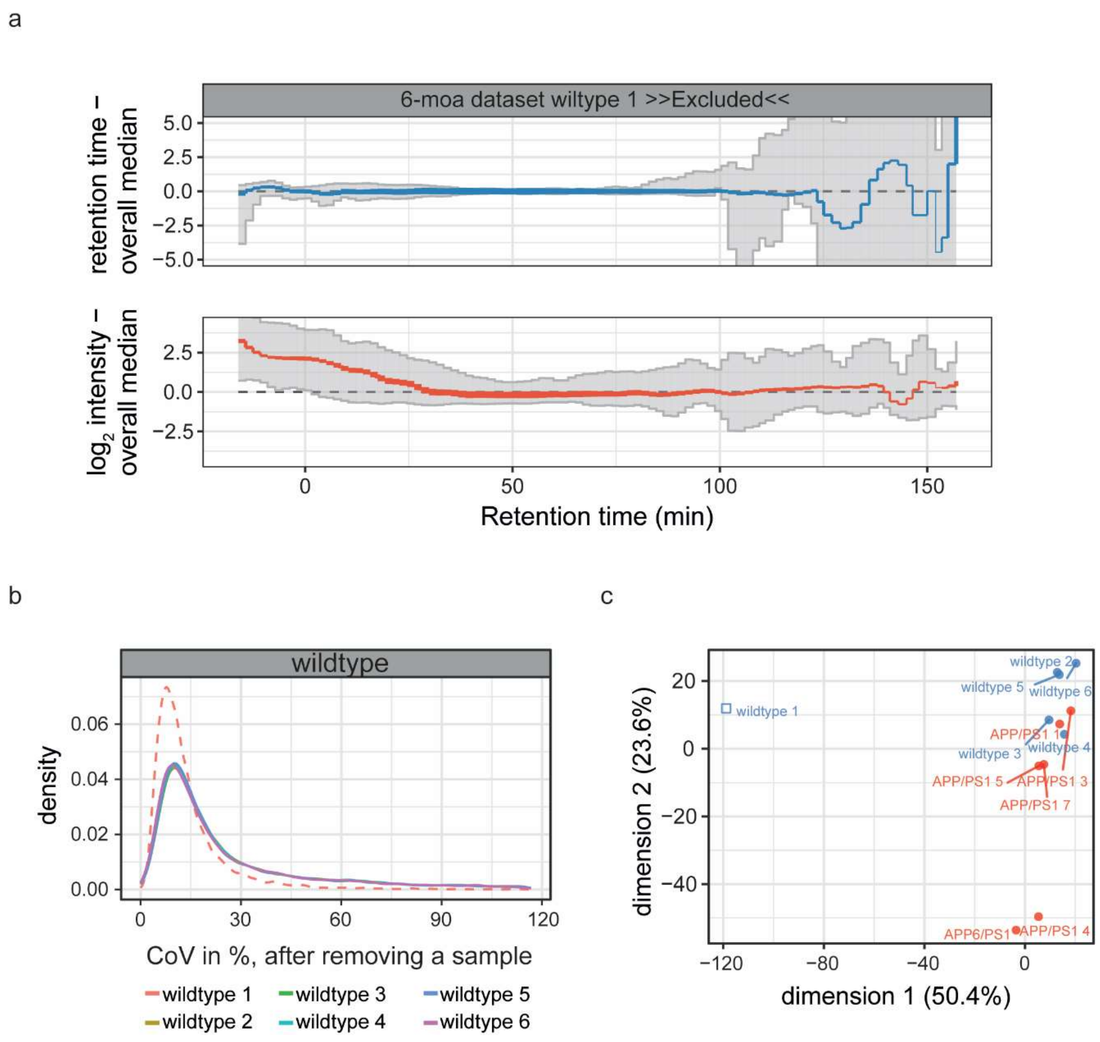

Figure S7. Quality control in MS-DAP of wildtype 1 in the 6-moa dataset. (**a**) Wildtype 1 showed large variation in retention time compared to the group median. Peptide retention time and abundance of wildype 1 are shown as a blue line (upper panel) and red line (lower panel), respectively, and are normalized to the median over all samples. Line widths correspond to the number of eluted peptides at that time point. The 5% and 95% quantiles are depicted in grey; (**b**) The effect of removing a sample prior to within-group Coefficient of Variation (CoV) computation is shown. The CoV is largely reduced after removal of wildtype 1; (**c**) A visualization of the first two Principal component analysis dimensions, showing that 50% of the variation explained by dimension 1 separates wildtype 1 from the other samples. Probabilistic Principal component analysis was performed on those peptides retained after filtering for differential expression analysis. The principal components and their respective percentage of variance explained are shown on the axis labels.

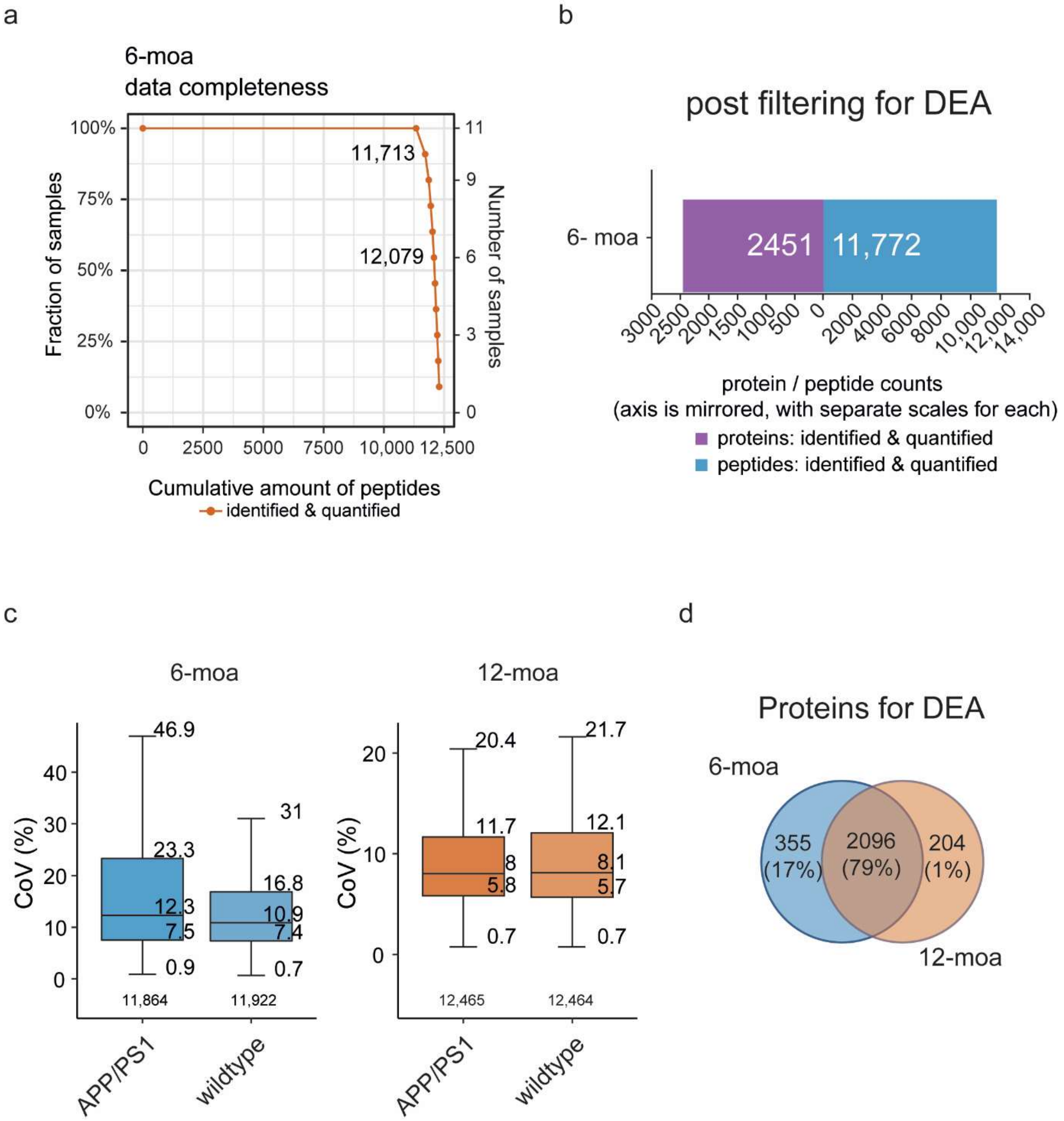

Figure S8. Quality control of the 6-moa dataset run against the directDIA library and comparison with the 12-moa dataset. (**a**) Cumulative distribution showing the number of peptides consistently identified across all samples; (**b**) Number of proteins and peptides that remain after filtering for differential expression analysis; (**c**) The Coefficient of Variation (CoV)s visualized as boxplots for the 6- and 12-moa datasets; (**d**) The number of proteins used for differential expression analysis observed in the 6- and 12-moa datasets.

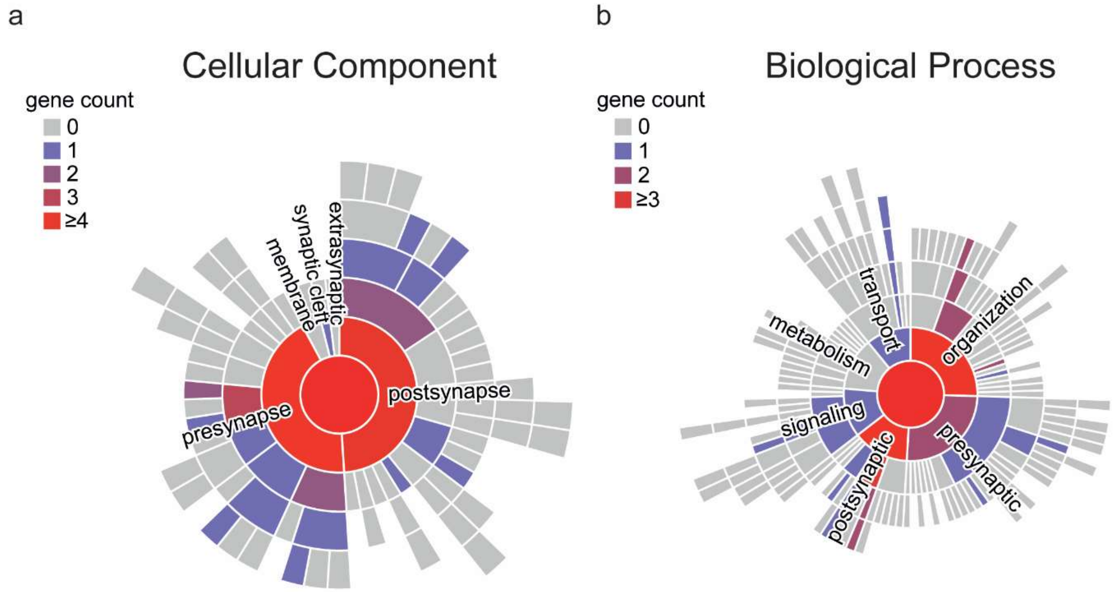

Figure S9. Sunburst plots of SynGO terms with highlighted gene counts of regulated 6- or 12-moa APP/PS1 mouse genes. The number of regulated genes annotated towards (**a**) location or (**b**) function terms are indicated per term.

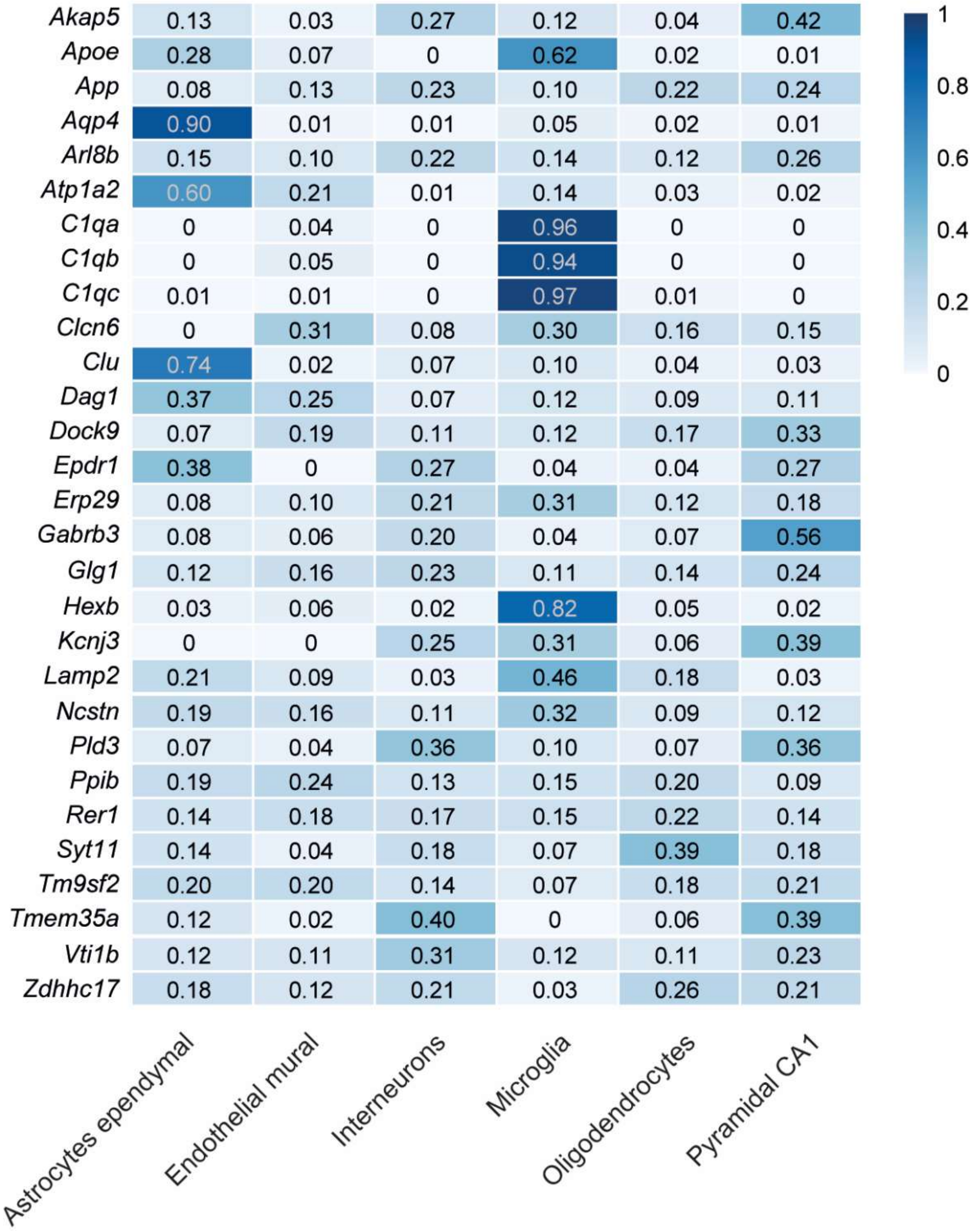

Figure S10. Gene expression cell-type specificity matrix of upregulated proteins in synaptosomes of 12-moa APP/PS1 mice. For each gene the expression taken from [1] is normalized to the total level in all cell-types combined, highlighting the relative distribution. Low expression is shown in white and high expression in dark blue.

Table S1. Regulated proteins that have been associated with AD in previous human studies.

Protein symbol	Association to human AD	References
APOE	Genome wide association study shows association of APOE loci with disease status	Jansen et al. 2019
APP	Segregation of genetic variant with familial AD observed in a linkage study	Goate et al. 1991
AQP4	Targeted analysis shows association between genetic AQP4 variants and cognitive decline after diagnosis of AD	Burfeind et al. 2017
C1Q(A-C)	Astrocyte derived exosomes of AD patients show increased levels of C1Q	Goetzl et al. 2018
	Increased levels of C1Q(A-C) have been observed in CSF and blood of AD patients, albeit inconsistently	Krance et al. 2019
CLU	Genome wide association study shows association of CLU loci with disease status	Jansen et al. 2019
LAMP2	Increased levels in CSF of AD patients observed with mass spectrometry	Sjödin et al. 2016
NCSTN	Targeted analysis shows segregation of genetic variant in the promoter region of NCSTN with disease status	Zhong et al. 2009
PLD3	Segregation of genetic variants with disease status using whole exome sequencing	Cruchaga et al. 2014

Table S2. SynGO annotations of regulated proteins at 6- or 12-moa APP/PS1 mice.

Gene symbol	GO term name	GO domain
AKAP5	regulation of postsynaptic neurotransmitter receptor endocytosis (GO:0099149)	Biological Process
AKAP5	postsynaptic density, intracellular component (GO:0099092)	Cellular Component
APOE	regulation of synapse organization (GO:0050807)	Biological Process
APOE	synaptic cleft (GO:0043083)	Cellular Component
APP	regulation of presynapse assembly (GO:1905606)	Biological Process
APP	presynaptic active zone (GO:0048786)	Cellular Component
APP	neuronal dense core vesicle (GO:0098992)	Cellular Component
ARL8B	regulation of anterograde synaptic vesicle transport (GO:1903742)	Biological Process
CLU	synapse (GO:0045202)	Cellular Component
DAG1	regulation of synapse organization (GO:0050807)	Biological Process
DAG1	regulation of neurotransmitter receptor localization to postsynaptic specialization membrane (GO:0098696)	Biological Process
DAG1	retrograde trans-synaptic signaling by trans-synaptic protein complex (GO:0098942)	Biological Process
DAG1	postsynaptic cytosol (GO:0099524)	Cellular Component
GABRB3	transmitter-gated ion channel activity involved in regulation of postsynaptic membrane potential (GO:1904315)	Biological Process
GABRB3	integral component of postsynaptic membrane (GO:0099055)	Cellular Component
GABRB3	integral component of postsynaptic specialization membrane (GO:0099060)	Cellular Component
GPC4	regulation of neurotransmitter receptor localization to postsynaptic specialization membrane (GO:0098696)	Biological Process
GPC4	synapse adhesion between pre- and post-synapse (GO:0099560)	Biological Process
GPC4	regulation of presynapse assembly (GO:1905606)	Biological Process
GPC4	synapse (GO:0045202)	Cellular Component
GPC4	anchored component of presynaptic membrane (GO:0099026)	Cellular Component
KCNJ3	voltage-gated ion channel activity involved in regulation of presynaptic membrane potential (GO:0099508)	Biological Process
KCNJ3	integral component of presynaptic membrane (GO:0099056)	Cellular Component
NCSTN	integral component of presynaptic membrane (GO:0099056)	Cellular Component
SYT11	regulation of synaptic vesicle endocytosis (GO:1900242)	Biological Process
SYT11	integral component of synaptic vesicle membrane (GO:0030285)	Cellular Component
SYT11	postsynapse (GO:0098794)	Cellular Component
SYT11	integral component of presynaptic active zone membrane (GO:0099059)	Cellular Component

Table S3. SynGO enrichment analysis of synaptic proteins regulated in 6- or 12-moa APP/PS1 mice using location terms.

Cellular Component

Ontology term	Gene count	p-value	q-value
presynaptic membrane	3	0.07	0.26
synapse	10	0.34	0.67
presynapse	5	0.50	0.67
postsynapse	4	0.73	0.73

Table S4. SynGO enrichment analysis of synaptic proteins regulated in 6- or 12-moa APP/PS1 mice using function terms.

Biological Process

Ontology term	Gene count	p-value	q-value
process in the postsynapse	4	0.09	0.17
regulation of postsynaptic membrane neurotransmitter receptor levels	3	0.08	0.17
process in the synapse	9	0.22	0.22
synapse organization	4	0.22	0.22

Reference

[1] A. Zeisel *et al.*, "Brain structure. Cell types in the mouse cortex and hippocampus revealed by single-cell RNA-seq.," *Science*, vol. 347, no. 6226, pp. 1138–1142, Mar. 2015, doi: 10.1126/science.aaa1934.
